# Functional connectivity dynamics reflect disability and multi-domain clinical impairment in patients with relapsing-remitting multiple sclerosis

**DOI:** 10.1016/j.nicl.2022.103203

**Published:** 2022-09-16

**Authors:** Amy Romanello, Stephan Krohn, Nina von Schwanenflug, Claudia Chien, Judith Bellmann-Strobl, Klemens Ruprecht, Friedemann Paul, Carsten Finke

**Affiliations:** a Department of Neurology, Charité – Universitätsmedizin Berlin, Corporate Member of Freie Universität Berlin, Humboldt-Universität zu Berlin, and Berlin Institute of Health, Berlin, Germany; b Berlin School of Mind and Brain, Humboldt-Universität zu Berlin, Berlin, Germany; c Experimental and Clinical Research Center, A Cooperation Between the Max Delbrück Center for Molecular Medicine in the Helmholtz Association and Charité – Universitätsmedizin Berlin, Germany; d Charité – Universitätsmedizin Berlin, Corporate Member of Freie Universität Berlin and Humboldt-Universität zu Berlin, Experimental and Clinical Research Center, Lindenberger Weg 80, 13125 Berlin, Germany; e Max Delbrück Center for Molecular Medicine in the Helmholtz Association (MDC), Berlin, Germany; f NeuroCure Clinical Research Center, Charité – Universitätsmedizin Berlin, Corporate Member of Freie Universität Berlin, Humboldt-Universität zu Berlin, and Berlin Institute of Health, Berlin, Germany; g Department of Psychiatry and Neurosciences, Charité – Universitätsmedizin Berlin, Corporate Member of Freie Universität Berlin, Humboldt-Universität zu Berlin, and Berlin Institute of Health, Berlin, Germany

**Keywords:** Multiple sclerosis, Functional connectivity, Resting state, Brain dynamics, Disability, Functional magnetic resonance imaging

## Abstract

**Background & Aim:**

Multiple sclerosis (MS) is an autoimmune disease of the central nervous system associated with deficits in cognitive and motor functioning. While structural brain changes such as demyelination are an early hallmark of the disease, a characteristic profile of functional brain alterations in early MS is lacking. Functional neuroimaging studies at various disease stages have revealed complex and heterogeneous patterns of aberrant functional connectivity (FC) in MS, with previous studies largely being limited to a static account of FC. Thus, it remains unclear how time-resolved FC relates to variance in clinical disability status in early MS. We here aimed to characterize brain network organization in early MS patients with time-resolved FC analysis and to explore the relationship between disability status, multi-domain clinical outcomes and altered network dynamics.

**Methods:**

Resting-state functional MRI (rs-fMRI) data were acquired from 101 MS patients and 101 age- and sex-matched healthy controls (HC). Based on the Expanded Disability Status Score (EDSS), patients were split into two sub-groups: patients without clinical disability (EDSS ≤ 1, n = 36) and patients with mild to moderate levels of disability (EDSS ≥ 2, n = 39). Five dynamic FC states were extracted from whole-brain rs-fMRI data. Group differences in static and dynamic FC strength, across-state overall connectivity, dwell time, transition frequency, modularity, and global connectivity were assessed. Patients’ impairment was quantified as custom clinical outcome z-scores (higher: worse) for the domains depressive symptoms, fatigue, motor, vision, cognition, total brain atrophy, and lesion load. Correlation analyses between functional measures and clinical outcomes were performed with Spearman partial correlation analyses controlling for age.

**Results:**

Patients with mild to moderate levels of disability exhibited a more widespread spatiotemporal pattern of altered FC and spent more time in a high-connectivity, low-occurrence state compared to patients without disability and HCs. Worse symptoms in all clinical outcome domains were positively associated with EDSS scores. Furthermore, depressive symptom severity was positively related to functional dynamics as measured by state-specific global connectivity and default mode network connectivity with attention networks, while fatigue and motor impairment were related to reduced frontoparietal network connectivity with the basal ganglia.

**Conclusions:**

Despite comparably low impairment levels in early MS, we identified distinct connectivity alterations between patients with mild to moderate disability and those without disability, and these changes were sensitive to clinical outcomes in multiple domains. Furthermore, time-resolved analysis uncovered alterations in network dynamics and clinical correlations that remained undetected with conventional static analyses, showing that accounting for temporal dynamics helps disentangle the relationship between functional alterations, disability status, and symptoms in early MS.

## Highlights

• Patients with early-MS show distinct alterations in functional connectivity dynamics.
• Multi-domain clinical outcome scores scale with disability status scores in patients.
• Temporal network dynamics vary with disability status in early MS.
• Frontoparietal–basal ganglia decoupling is a novel, clinically-relevant signature of MS.

## 1 Introduction

Identifying aberrant signatures of brain activity in multiple sclerosis (MS) is a key research target for improving our understanding of the complex interplay between symptom severity and functional network behavior, especially in early MS. However, the characterization of common and clinically relevant functional alterations has been challenging, evidenced by a growing body of literature reporting complex, and occasionally, contradictory patterns of functional brain changes in MS.

Recent studies highlight subcortical brain regions as key players in the functional changes seen in MS: altered functional connectivity (FC) of the thalamus (Lin et al., 2019, Hidalgo de la Cruz et al., 2018, Tona et al., 2014) and basal ganglia (BG) (Finke et al., 2015, Tijhuis et al., 2021) has been consistently linked to cognitive function and fatigue. In contrast, while many studies implicate default mode network (DMN) dysfunction in MS, the directionality and thus the interpretation of this dysfunction remains inconsistent: both increased (Hawellek et al., 2011, Meijer et al., 2017) and opposingly, decreased DMN connectivity (Rocca et al., 2018) have been identified in the relationship between functional alterations and cognitive impairment in MS.

Potential explanations for such heterogeneity include the temporally dynamic evolution of the disease, the ensuing variance in disease severity and disability, its diverse cognitive and behavioral phenotypes as well as methodological variability across functional MRI (fMRI) studies. Furthermore, much previous research has been limited to a static account of brain activity in which FC is computed on signals over the entire scan duration. However, recent advances in time-resolved approaches – where FC is computed on a finer temporal scale and clustered into recurrent connectivity states – have made substantial contributions to identifying functional brain signatures in various states of health (Allen et al., 2014) and disease (Damaraju et al., 2014, Demirtaş et al., 2016, von Schwanenflug et al., 2022).

Furthermore, evidence from recent investigations into the hierarchical organization of brain dynamics in healthy populations has revealed a principled temporal gradient in which networks operate (Vidaurre et al., 2017). This gradient from primary sensorimotor systems to higher-order cognitive systems is thought to reflect the variance in timescales of information encoding speed and activity across networks (Raut et al., 2020). These differences further highlight the need for continued research into brain dynamics using time-varying techniques, which more sensitively assess moment-to-moment changes that are not detectable with static approaches.

Such time-resolved techniques have recently highlighted reduced network centrality (Eijlers et al., 2019) and FC dynamics (d’Ambrosio et al., 2020) in cognitively impaired MS patients, as well as subtype-specific altered FC and associations with cognitive and motor impairment (Hidalgo de la Cruz et al., 2021). While dynamic whole-brain connectivity studies thus represent a promising avenue to unravel the relationship between functional alterations and clinical outcomes, it remains unclear how altered network dynamics relate to disability status, disease severity, and the multi-domain impairments commonly observed in MS.

Here, we characterize the spatiotemporal patterns of FC alterations in a large sample of patients with early relapsing-remitting MS (RRMS). To this end, we investigate both static and dynamic FC alterations compared to healthy participants and relate these functional dynamics to disability status and clinical impairment across multiple domains including cognitive, behavioral, and affective scores as well as structural brain measures.

## 2 Materials & methods

### 2.1 Study participants and clinical assessment of patients

Patients were recruited from the outpatient clinic of the Clinical and Experimental Multiple Sclerosis Research Center at Charité - Universitätsmedizin Berlin from 2014 to 2018. At the time of their study visit, all patients fulfilled the McDonald criteria (Thompson et al., 2018) for an RRMS diagnosis (Table 1). Patients did not have any comorbid neurological or psychiatric conditions. Healthy control participants (HC) were recruited using the Charité intranet and were without neurological or psychiatric conditions.

**Table 1:** Demographic information for patients and healthy control participants.

	**HC**	**Patients (Whole sample)**	**Patients (EDSS ≤ 1)**	**Patients (EDSS ≥ 2)**	**p**^a^
**N**	101	101	36	39	
**Sex (F/M)**	67/34	67/34	20/16	27/12	
**Age (Mean ± SD years)**	35.04 ± 10.33	36.11 ± 10.33	31.55 ± 6.12	40.21 ± 11.64	< 0.001*
**EDSS (Median (IQR))**	NA	1.5 (1.5)	1 (1)	2.5 (1)	< 0.001*
**Disease duration (Mean ± SD years)**	NA	7.07 ± 7.99	4.24 ± 3.46	10.86 ± 10.49	< 0.001*
**MS-specific Medication (n)** If yes, which? (n)	NA	Yes (70)	Yes (23)	Yes (31)	
		No (27)	No (13)	No (8)	
		Not reported (4)			
		Interferon beta (10)	Interferon beta (4)	Interferon beta (4)	
		Glatiramer acetate (20)	Glatiramer acetate (9)	Glatiramer acetate (8)	
		Fumarate (15)	Fumarate (6)	Fumarate (4)	
		Azathioprine (1)	Natalizumab (1)	Azathioprine (1)	
		Natalizumab (4)	Fingolimod (2)	Natalizumab (2)	
		Fingolimod (11)	Other (1)	Fingolimod (6)	
		Teriflunomide (3)		Teriflunomide (3)	
		Other (6)		Other (3)	

*HC: healthy control participants; MS EDSS ≤ 1: MS patients with Expanded disability status scale score<1; MS EDSS ≥ 2: MS patients with Expanded disability status scale score greater than 2; SD: standard deviation; EDSS: Expanded disability status scale; IQR: interquartile range; NA: not applicable*.

a p-value corresponding to permutation-based null estimation of *T*-statistic between MS patients with EDSS≤1 and MS patients with EDSS≥2.

Patients underwent a complete neurological assessment and were evaluated by a board-certified neurologist. Clinical and neuropsychological evaluation occurred on the same day or within one day of MRI scan acquisition. A patient’s current level of disability was evaluated using the Expanded Disability Status Scale (EDSS). Motor performance speed was assessed using the Timed 25-Foot Walk Test and the Nine-Hole Peg Test. Visual acuity was assessed using both the Early Treatment Diabetic Retinopathy Study (ETDRS) and Sloan scales at high, medium, and low contrasts.

### 2.2 Neuropsychological assessment

Patients also underwent an extensive battery of neuropsychological tests. Fatigue was assessed using the Fatigue Severity Scale (FSS). Depressive symptoms were assessed using the Beck’s Depression Inventory II (BDI-II). It is important to note that, while there was considerable variance in depressive symptoms, average BDI-II raw scores did not suggest manifest clinical depression.

Cognitive performance was assessed using the Brief Repeatable Battery of Neuropsychological Tests (BRB-N), including the following sub-tests: the Selective Reminding Test (SRT) as a measure of verbal learning and memory, the 10/36 Spatial Recall Test (SPAT) as a measure of visuospatial memory, the Symbol Digit Modalities Test (SDMT) as a measure of processing speed and attention, the three-second version of the Paced Auditory Serial Addition Test (PASAT) as a measure of information processing speed and calculation ability, and the Word List Generation Test (WLG) as a measure of verbal fluency.

### 2.3 Group comparisons by disability scores and clinical rationale

Age- and sex-matching of patients and HCs was performed using a custom matching algorithm. Thus, 101 patients and 101 matched HCs were initially included; 92 patients had an EDSS rating from the date of scan acquisition. For the purposes of group comparisons, patients were split into subgroups of mild to moderate disability (EDSS ≥ 2, n = 39) and no disability (EDSS ≤ 1, n = 36) using the upper and lower 30th percentile threshold. Seventeen patients with an EDSS score of 1.5 were thus excluded because their score corresponded exactly to the median of the distribution across patients. Patients with EDSS ≥ 2 were significantly older and had a longer disease duration than patients with EDSS ≤ 1 (Table 1). The final sample used for statistical analysis of group differences thus included 75 patients and 75 HCs. Additional details on the matching procedure and the patient cohort split are provided in supplemental material (SM) sections 1.1 and 1.2.

Patients in the “no disability” subgroup are those with an EDSS rating of 0 or 1. These are patients with either a normal neurological exam (EDSS = 0) or those with no disability but minimal signs of impairment in only one of the functional systems evaluated within the EDSS assessment (EDSS = 1). Overall, these patients are characterized by the absence of clinical disability (Kurtzke, 1983). Patients in the “mild to moderate disability” subgroup are those with an EDSS rating of 2 or greater. Although the range of EDSS ratings in this group was 2 through 5.5, the median EDSS was 2.5 (IQR = 1); no patient had a score of 4.5 or 5, and only one patient had a score of 5.5 (Supplementary Fig. 2). Thus, this group of patients showed minimal to moderate levels of disability in one or more functional systems incorporated in the EDSS assessment (Kurtzke, 1983).

### 2.4 MRI acquisition

MRI data were acquired at the Berlin Center for Advanced Neuroimaging at Charité-Universitätsmedizin Berlin, Germany, using a 20-channel head coil on a 3 T Trim Trio scanner (Siemens, Erlangen, Germany). For each participant, the following sequences were acquired and used in the present study: a 10-minute resting-state-fMRI (rs-fMRI) scan was collected using a repetition time (TR) of 2250 ms (TE = 30 ms, 260 volumes, matrix size = 64 × 64, 37 axial slices, slice thickness = 3.4 mm, voxel size = 3.4 × 3.4 × 3.4 mm^3^); a T1-weighted structural scan was acquired using a magnetization-prepared rapid gradient echo (MPRAGE) sequence (1 mm^3^ isotopic resolution, matrix size = 240 × 240, 176 slices); and a T2-weighted fluid-attenuated inversion recovery (FLAIR) sequence was acquired with a TR of 5000 ms (TE = 388 ms, FOV = 250 × 250 mm^2^, matrix size 250 × 250, 76 slices, slice thickness = 1 mm, 1 mm isotopic resolution). All participants gave written informed consent, and the study was approved by the local ethics committee.

### 2.5 Conventional MRI analysis

T2-hyperintense brain lesion segmentation was performed manually using ITK-SNAP by two MRI technicians with more than 10 years of experience in MS research. The Functional Magnetic Resonance Imaging of the Brain (FMRIB) Software Library (FSL) “cluster” and “fslstats” tools were used to calculate lesion volumes and counts. Lesion load was taken as a patient’s total lesion volume in milliliters. Total normalized brain volume was calculated from lesion-filled MPRAGE scans using FSL SIENAX.

### 2.6 Calculation of multi-domain clinical outcome z-scores

To systematically assess the relationship between functional brain measures and variance across the patient cohort in neuropsychological, behavioral, and structural MRI measures, we computed a custom set of domain-specific clinical outcome scores. Individual test scores and structural MRI measures were grouped into the following seven domains: cognition, motor, vision, depression, fatigue, lesion load and brain volume. Table 2 shows a detailed list of measures assigned to each domain. For each domain, a z-score was calculated according to the following procedure: the summary or total score for each test was calculated across its items according to the respective test manual; the test’s z-score was then computed across all patient scores; for domains that included multiple tests, a composite z-score was computed by averaging across all tests assigned to that domain. Thus, each patient ultimately obtained a single value for each domain. Please note, to account for directionality differences, test scores for vision, cognition, and brain volume were re-coded by multiplying by −1. Thus, all domain z-scores represent impairment indices, where higher values signify worse performance, more severe symptoms, or higher brain atrophy, respectively.

**Table 2:** Domain assignments for selected neuropsychological, clinical, and structural MRI measures.

Domain	Measures	Details
Vision	ETDRS 100 % high contrast	Right eye, decimal acuity
		Left eye, decimal acuity
		Right eye, number of letters correct
		Left eye, number of letters correct
	ETDRS 10 % medium contrast	Right eye, decimal acuity
		Left eye, decimal acuity
		Right eye, number of letters correct
		Left eye, number of letters correct
	Sloan 2.5 % low contrast	Right eye, decimal acuity
		Left eye, decimal acuity
		Right eye, number of letters correct
		Left eye, number of letters correct
	Sloan 1.25 % low contrast	Right eye, decimal acuity
		Left eye, decimal acuity
		Right eye, number of letters correct
		Left eye, number of letters correct
Motor	Timed 25-Foot Walk Test (T25-FW)	
	Nine-Hole Peg Test (9-HPT)	
Fatigue	Fatigue severity scale (FSS)	
Depression	Beck’s depression inventory II (BDI-II)	
Cognition	Selective reminding test – long-term storage (SRT-LR)	
	Selective reminding test – consistent long-term retrieval (SRT-CLTR)	
	Selective reminding test – delayed recall (SRT-DR)	
	Spatial recall test (SPAT)	
	Spatial recall test – delayed recall (SPAT-DR)	
	Symbol digit modality test (SDMT)	
	Paced auditory serial addition test (PASAT)	
	Word list generation test (WLG)	
Brain volume	Total normalized brain volume	
Lesion load	T2-FLAIR total lesion volume	

Table includes domain name, measures or questionnaires assigned to each domain, and details of a given measure, where appropriate. *ETDRS: Early Treatment Diabetic Retinopathy Study*.

### 2.7 Resting-state fMRI analysis

#### 2.7.1 Preprocessing

All analyses were performed with MATLAB (R2019b) and R (3.6.3). Preprocessing was performed using the CONN toolbox (version 18.b; (Whitfield-Gabrieli and Nieto-Castanon, 2012). The following preprocessing steps were applied using each participants’ rs-fMRI and structural scans, in line with the default settings of the CONN toolbox: discarding of first five volumes of the rs-fMRl scan to account for scanner gradient stabilization, realignment to the first volume using b-spline interpolation, slice-timing correction using sinc-interpolation to time-shift and resample functional data slices, direct tissue segmentation and spatial normalization to standard Montreal Neurological Institute space including resampling of functional data to 2 mm isotropic voxels and structural data to 1 mm isotropic voxels, and finally, spatial smoothing by convolving with a Gaussian kernel of 8 mm full width at half maximum.

#### 2.7.2 Group independent component analysis (GICA)

The “spatial GICA” feature of the Group ICA of fMRI toolbox (GIFT v4.0b, http://mialab.mrn.org/software/gift/index.html) was then used to reduce all participants' preprocessed rs-fMRI data into 100 group spatial independent components. To this end, each participant's 4D-functional data (255 volumes) were reduced to 150 temporal principal components (PC) that were maximally independent. All participant components were then concatenated, and a second principal component analysis (PCA) was performed on the whole-sample matrix, resulting in 100 PCs. From this PCA matrix, the *Infomax* algorithm (Calhoun et al., 2001) for ICA was used to extract 100 group independent components (ICs), which were then checked for stability in *ICASSO* (Himberg and Icasso, 2003) by performing 20 iterations of the ICA algorithm. Lastly, for each participant, a set of 100 component spatial maps and corresponding component time-courses were extracted with the spatiotemporal regression back-reconstruction algorithm (Du and Fan, 2013) in GIFT. This method has been extensively described in (Allen et al., 20142014, Calhoun et al., 2001).

#### 2.7.3 Component rating and network assignment

Group ICs were manually classified as signal or noise based on criteria recommended in (Griffanti et al., 2017). This was performed by two independent raters (AR, NS), and any disagreement was resolved through rating by a third independent rater (CF). Forty-seven group ICs were classified as “signal”, while 63 were discarded as “noise”. Signal components were assigned to the following cortical resting-state networks (RSN) according to (Yeo et al., 2011): ventral attention (vATT), dorsal attention (dATT), somatomotor (SMN), DMN, visual (VIS), and frontoparietal (FPN). Upon visual inspection, components overlapping subcortical (SC) and cerebellar (CB) brain regions were assigned to these groups for subsequent analyses. The SC cluster contained only one component which corresponded to the bilateral basal ganglia (BG). Thus, this component will henceforth be referred to as “BG”. Additional details on component rating are provided in SM section 1.3.

#### 2.7.4 Functional network analysis

Using the “temporal dFNC” toolbox of GIFT, participants’ component time-courses underwent additional steps of nuisance regression including despiking, detrending, regression of realignment parameters and derivatives, and bandpass filtering (0.01–0.15 Hz) – in line with default settings of the toolbox. Static FC was computed using pairwise Pearson’s correlations across all time-points. Dynamic FC was performed using sliding window correlations, as described by (Allen et al., 2014), with a window size of 22 TR and a slide length of 1TR. Raw correlation coefficients were Fisher-Z transformed. K-means clustering was employed to extract distinct connectivity states using the “city-block” distance metric, as recommended for high-dimensional data (Aggarwal et al., 2001). Based on convergence between the elbow criterion and Dunn’s Index (Dunn, 1974), we identified five states. For reproducibility purposes, the numerical identity of the clusters was re-coded according to descending total occupancy across the sample of participants. For each participant, the median over all dFC windows spent in a state was computed for further analyses. Additional details are provided in SM sections 1.4–5.

#### 2.7.5 Dynamic metrics

We calculated the following additional measures of state dynamics: mean dwell time (the mean number of windows spent in a state upon entering it), fraction time (i.e., fractional occupancy; the proportion of scan time spent in a state), transition frequency (the number of switches between each pair of states), and state stickiness (the number of times a participant remained in the same state over two consecutive windows). These dynamic metrics have previously been defined and used to characterize connectivity states in (Allen et al., 20142014, von Schwanenflug et al., 2022).

State-wise average connectivity and modularity were calculated as measures of global connectivity and topology, respectively. Modularity was calculated using the community Louvain algorithm of the Brain Connectivity Toolbox (2019–03-03) (Rubinov and Sporns, 2010). Average connectivity was calculated as the global mean connectivity strength over all windows spent in that state. Additional details are provided in SM section 1.6.

#### 2.7.6 Across-state overall connectivity (ASOC)

To explore the influence of differences in fraction time on a summary measure of global brain connectivity, we computed an additional “across-state overall connectivity” (ASOC) metric. This was calculated by averaging across a participant’s dFC data from all windows, resulting in one grand-average value across all connections and states, as well as 7 intra-network ASOC values (not applicable to BG which had only one component), and 8 inter-network ASOC values. Inter-network ASOC values were computed as the overall connectivity between one RSN and the rest of the brain.

### 2.8 Analysis of group differences

Between-group differences for static and dynamic FC were computed using a “non-parametric” two-sample T-test that estimates the null distribution by permuting group labels (see https://version.aalto.fi/gitlab/BML/bramila). For static FC and for each dFC state, differences were assessed between three group pairs: HC vs patients with EDSS ≤ 1, HC vs patients with EDSS ≥ 2, and patients with EDSS ≤ 1 vs patients with EDSS ≥ 2. Statistical significance was defined at an alpha level of 0.05 after false discovery rate (FDR) correction for multiple comparisons according to (Benjamini and Hochberg, 1995). For static FC, each participant had a vector of 1081 values (lower triangle of the symmetrical 47-by-47 component matrix). For dynamic FC, each participant had a vector of 1081 values for each of the five connectivity states. We used a mass univariate statistical approach to test the hypothesis that for each of the 1081 FC values, group 1 differed from group 2. For FDR correction in the case of static FC, we considered a family of univariate tests to be within a group pair (e.g., HC vs EDSS ≤ 1) and thus correction was applied over these 1081 tests. In the case of dynamic FC, we considered a family of univariate tests to be within a group pair *and* within a state – thus FDR correction was applied over 1081 tests for each state and each between-group comparison.

Group differences in dynamic metrics were assessed using a within-state approach. Linear regression was used to remove age-related variance. Using the model residuals, Kruskal-Wallis (KW) omnibus tests on the three groups were performed. If the null hypothesis was rejected, post-hoc pairwise comparisons were performed using Dunn’s tests with FDR correction. In the case of temporal dynamic metrics, we considered a family of tests to be within a state and within a measure (e.g., dwell time) – thus, FDR correction was applied over three tests (e.g., state 1 dwell time across HC, patients with EDSS ≤ 1, and patients with EDSS ≥ 2).

To test group differences in ASOC, an identical approach to the dynamic metric analyses was applied. In the case of group effects on inter-network ASOC (e.g., between the FPN and the rest of the brain), an additional analysis was performed between each pair of RSNs to identify which connections were driving the effect.

### 2.9 Correlation analyses

Group differences in domain clinical outcome scores were evaluated using non-parametric permutation tests. FDR correction was applied over seven hypothesis tests, in that patients with mild to moderate disability would differ from patients with no disability in each of the several clinical domains. Spearman’s partial correlations were calculated between FC values and clinical scores, controlling for age. In the case of dynamic FC, a total of 37,835 correlations were tested (1081 FC values × 7 domains × 5 states). FDR-correction was applied within a state and within a domain, thus correcting over 1081 tests in each case. For correlations between dynamic metrics, ASOC, and clinical scores, Spearman’s correlations were computed using regression model residuals.

## 3 Results

### 3.1 Clinical characterization

**Fig. 1:**
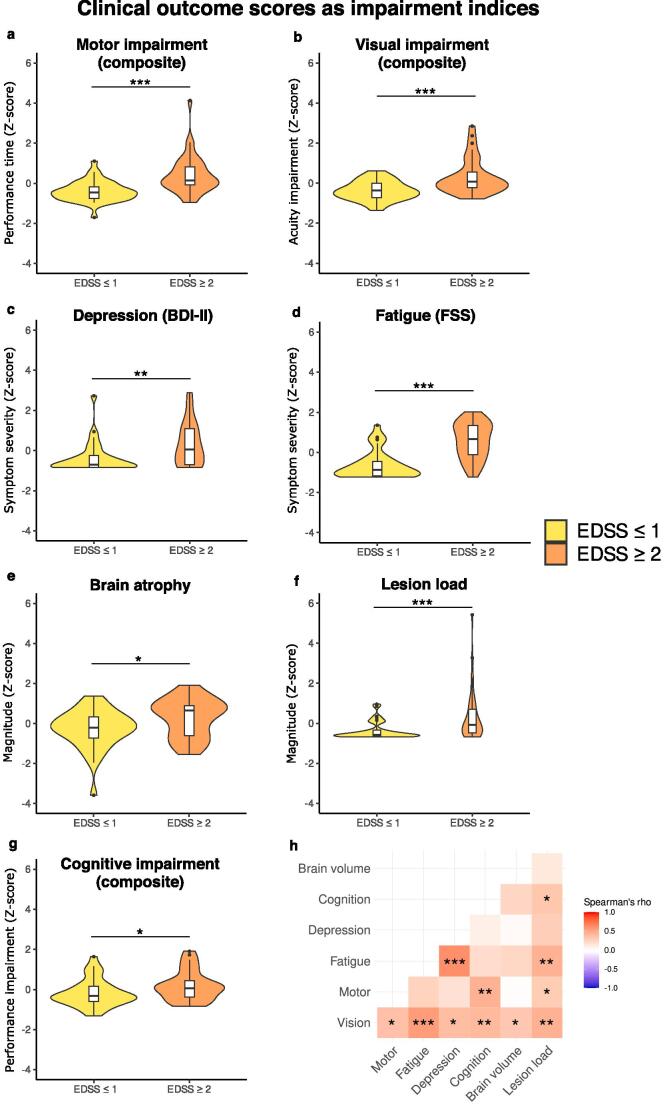
Between-group differences in clinical outcome z-scores across domains. Panels a-g: violin plots show distributions of clinical outcome scores in patients with mild to moderate disability (orange) and those without disability (yellow); Motor, vision, and cognitive symptoms domains are composite scores (see Table 2 for details on individual tests in each domain). Please note, all clinical outcome z-scores are coded such that higher values represent higher impairment (i.e., worse performance, higher symptom severity, or more atrophy). Patients with mild to moderate levels of disability in terms of Expanded disability status scale (EDSS) rating also showed higher scores in all other domains compared to patients with no disability. Panel h: correlation matrix where cells contain Spearman’s correlation coefficients between each pair of clinical outcome scores, computed across all patients. Asterisks indicate significance level: * = p_FDR_ < 0.05, ** = p_FDR_ < 0.01, *** = p_FDR_ < 0.001. (For interpretation of the references to color in this figure legend, the reader is referred to the web version of this article.)

Patients with mild to moderate disability (EDSS ≥ 2) showed significantly worse outcomes across all clinical domains compared to patients without disability (EDSS ≤ 1), including worse cognitive and motor performance, worse visual acuity, higher depressive and fatigue symptoms, as well as higher lesion load and total brain atrophy (Table 3, Fig. 1). Additionally, rank-based correlation analyses confirmed a significant positive relationship between disability ratings according to EDSS and the distribution of clinical outcome scores across patients (Supplementary Table 1, Supplementary Fig. 3), and this held true across all impairment domains.

**Table 3:** Group differences in domain-specific clinical outcome z-scores.

**Domain**	**N (MS EDSS ≤ 1)**	**N (MS EDSS ≥ 2)**	**Mean ± SD (MS EDSS ≤ 1)**	**Mean ± SD (MS EDSS ≥ 2)**	**p_FDR_**	**T**
Vision	29/36	35/39	−0.38 ± 0.46	0.31 ± 0.86	< 0.001*	−3.987
Motor	36/36	38/39	−0.39 ± 0.51	0.40 ± 0.94	< 0.001*	−4.534
Fatigue (FSS)	33/36	36/39	−0.63 ± 0.68	0.58 ± 0.87	< 0.001*	−6.347
Depression (BDI-II)	29/36	33/39	−0.37 ± 0.75	0.32 ± 1.06	0.004*	−2.918
Cognition	34/36	33/39	−0.19 ± 0.66	0.17 ± 0.72	0.023*	−2.097
Total brain atrophy	33/36	32/39	−0.28 ± 0.96	0.28 ± 0.94	0.014*	−2.345
Lesion load	35/36	39/39	−0.36 ± 0.43	0.32 ± 1.22	< 0.001*	−3.232

Table includes domain name, number of patients per group with complete data, group mean and standard deviation of computed Z-score, FDR-corrected p-value, and test statistic (T). *MS EDSS ≤ 1: MS patients with expanded disability status scale score less than or equal to 1; MS EDSS ≥ 2: MS patients with expanded disability status scale score greater than or equal to 2; FDR: false discovery rate; FSS: Fatigue severity scale; BDI-II: Beck’s depression inventory – second edition.*

### 3.2 Functional network analysis

#### 3.2.1 Static FC group differences

**Fig. 2:**
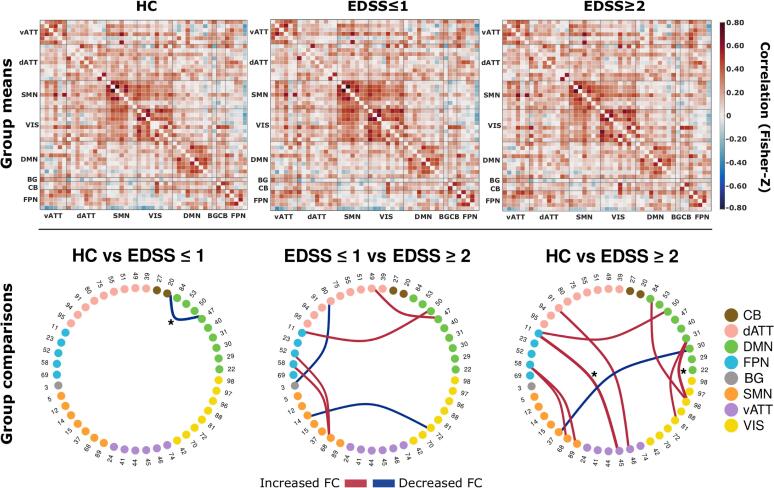
Whole-brain static functional connectivity matrices and between-group differences. Top: Mean static FC matrices computed by taking the average across all participants within a group. Pearson correlation coefficients were Fisher-Z transformed. Abbreviations on x- and y-axes indicate connections grouped by corresponding resting state networks. Matrix diagonals were zeroed for visualization purposes. Bottom: Between-group comparison results from the relevant permutation-based T-test. Group pairs used for comparison are denoted above each ring graph. Nodes and adjacent numbers identify the corresponding component (see supplementary material seciont 1.3 for detailed component information). Nodes are grouped and color coded according to RSNs. Edge width maps the magnitude and direction of *T*-statistic. Edges were thresholded at an uncorrected p-value of 0.001. Asterisks indicate tests with a false discovery rate adjusted p-value < 0.05.

Group mean sFC and between-group comparisons are shown in Fig. 2. Here, comparisons between patients with mild to moderate disability and matched HCs revealed decreased FC between the DMN and cerebellum in patients. Patients with mild to moderate disability showed a pronounced pattern of inter-network FC alterations compared to matched HCs, predominantly with FC increases between the DMN and FPN to other RSNs. In comparisons between both patient groups, patients with mild to moderate disability showed increased sFC between the FPN, SMN, and DMN and reduced connectivity between VIS-SMN and BG-dATT compared to patients without disability. However, these latter findings were not significant after FDR correction (Table 4).

**Table 4:** Between-group differences in component-wise static functional connectivity.

**Group comparison**	**Regions**^a^	**Networks**	**Components**	**p_uncorr_**	**p_FDR_**	**T**	**d**
HC vs EDSS ≤ 1	ANG (left) – CB	DMN – CB	47 – 20	< 0.001	0.030*	4.043	0.547
HC vs EDSS ≥ 2	Frontal pole **–** PCG	FPN – vATT	11 – 45	< 0.001	< 0.001*	−4.429	−0.831
	MFG **–** ACG	dATT – vATT	91 – 46	< 0.001	0.088	−3.617	−0.569
	STG **–** Precentral gyrus	DMN – SMN	30 – 37	< 0.001	0.088	3.513	0.646
	MFG (right) **–** SMA	FPN – SMN	58 – 68	< 0.001	0.088	−3.286	−0.622
	MFG (right) **–** Postcentral gyrus	FPN – SMN	58 – 89	< 0.001	0.065	−3.564	−0.621
	ANG (right) **–** Lingual gyrus	DMN – VIS	31 – 81	< 0.001	0.088	−3.278	−0.638
	ANG (right) **–** LOcc	DMN – VIS	31 – 96	< 0.001	< 0.001*	−4.362	−0.759
	Precuneus cortex **–** LOcc	DMN – VIS	84 – 96	< 0.001	0.088	−3.481	−0.713
	Frontal pole **–** Frontal pole	DMN – FPN	50 – 11	< 0.001	0.088	−3.410	−0.657
EDSS ≤ 1 vs EDSS ≥ 2	ANG (left) **–** SMG	DMN – dATT	47 – 49	< 0.001	0.155	−3.619	−0.642
	BG **–** Superior parietal lobule (right)	BG – dATT	3 – 80	< 0.001	0.155	3.414	0.430
	Lingual gyrus **–** Precentral gyrus (left)	VIS – SMN	72 – 14	< 0.001	0.155	3.358	0.861
	MFG (left) **–** SMA	FPN – SMN	52 – 68	< 0.001	0.155	−3.204	−0.617
	MFG (right) **–** SMA	FPN – SMN	58 – 68	< 0.001	0.155	−3.422	−0.675
	Frontal pole **–** Frontal pole	FPN – DMN	11 – 50	< 0.001	0.155	−3.476	−0.724

Table includes an indication of group pair used for a set of tests (Group comparison), brain region (Regions) and network labels (Networks) for corresponding components (Components), uncorrected p-values (p_uncorr._), FDR-corrected p-values (p_FDR_), *T*-values and Cohen’s effect size (d). *EDSS: Expanded disability status scale; FDR: false discovery rate; ANG: angular gyrus; CB: cerebellum; PCG: paracingulate gyrus; MFG: middle frontal gyrus; ACG: anterior cingulate gyrus; STG: superior temporal gyrus, posterior division; SMA: supplementary motor area; LOcc: Lateral occipital cortex, superior division; SMG: supramarginal gyrus; BG: basal ganglia; DMN: default mode network; FPN: fronto-parietal control network; vATT: ventral attention network; dATT: dorsal attention network; SMN: somatomotor network; VIS: visual network.*

a Component region labels are bilateral unless otherwise noted.

#### 3.2.2 Dynamic FC states and group differences in dynamic connectivity strength

**Fig. 3:**
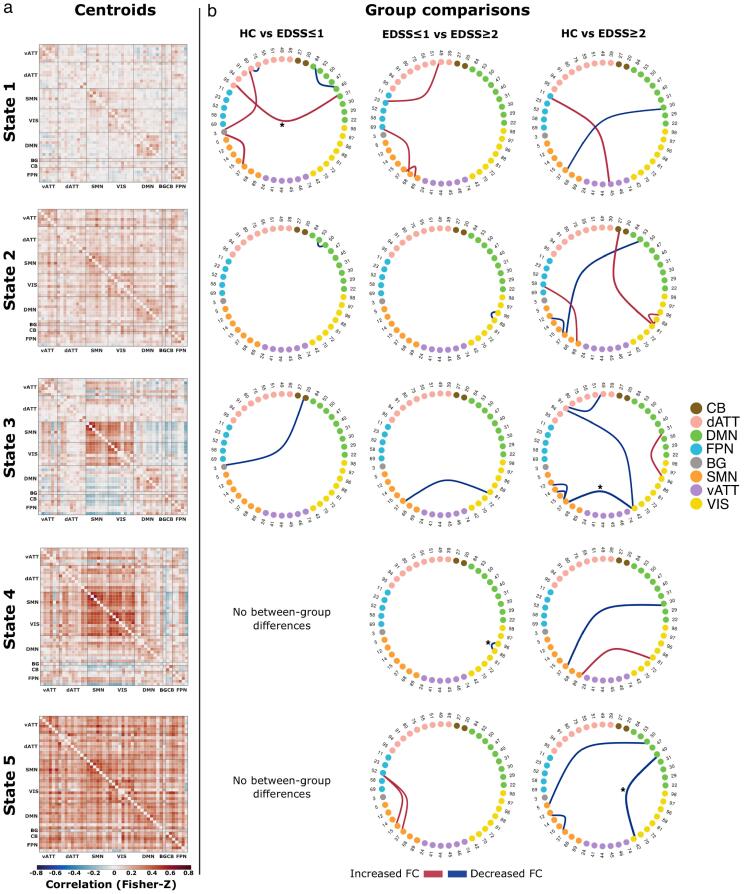
Whole-brain dynamic state centroids and between-group differences. Panel a: State centroid positions output from k-means clustering algorithm. Matrix diagonals were zeroed for visualization purposes. Panel b: Between-group comparison results from permutation-based T-tests. Group pairs used for comparisons are denoted above each column of ring graphs. Nodes and adjacent numbers identify the corresponding component (see supplemental material section 1.3 for detailed component information). Nodes are grouped and color coded according to their RSN assignment. Edge width maps the magnitude and direction of *T*-statistic. Edges were thresholded at an uncorrected p-value of 0.001. Asterisks indicate tests with an FDR-corrected p-value < 0.05.

Fig. 3 shows the centroid position of each connectivity state and results of between-group comparisons. State 1 was the most frequently occurring and least globally connected state, while state 5 was the least frequently occurring state with highest global connectivity and lowest modularity. We observed an inverse relationship between the distributions of global average connectivity and modularity across states, as expected from previous network studies (Krohn et al., 2021). Additional state characteristics are shown in SM section 2.1.

State-wise between group comparisons revealed a widespread pattern of FC alterations. Compared to HCs, patients with EDSS ≤ 1 had altered FC in three states – predominantly involving regions of the DMN and dATT. Interestingly, patients with EDSS ≥ 2 compared to HCs, showed a more spatially widespread and temporally persistent pattern of altered FC, implicating all connectivity states. Alterations were found in all five states and predominantly involved the VIS and SMN, as well as DMN, dATT, and FPN to a lesser extent. Comparisons between patient subgroups likewise revealed altered FC in all five states. In states 2–4, patients with EDSS ≥ 2 exhibited reduced FC with the VIS. Additionally, patients with EDSS ≥ 2 exhibited increased FC in states 1 and 5. This predominantly involved connections between the FPN-SMN and FPN-dATT. However, these latter increases were not significant after FDR correction (Table 5, Table 6, Table 7).

**Table 5:** Dynamic functional connectivity results for comparison between healthy participants and patients with EDSS ≤ 1.

**State**	**Regions**^a^	**Networks**	**Components**	**p_uncorr._**	**p_FDR_**	**T**	**d**
1	Superior parietal lobule (right) – Superior parietal lobule (left)	dATT – dATT	80 – 75	< 0.001	0.089	3.527	0.930
	BG – Superior parietal lobule (right)	BG – dATT	3 – 80	< 0.001	0.171	−3.376	−0.729
	ANG (right) – LOcc (left)	DMN – dATT	31 – 95	< 0.001	0.050*	−3.741	−0.941
	BG – Precentral gyrus	BG – SMN	3 – 37	< 0.001	0.075	−3.929	−0.649
	Precuneus cortex – Precuneus cortex	DMN – DMN	84 – 40	< 0.001	0.089	3.593	0.645
2	ANG (right) – Frontal pole	DMN – DMN	53 – 50	< 0.001	0.303	3.451	0.764
3	CB – BG	CB – BG	20 – 3	< 0.001	0.501	3.456	0.709

Table includes state labels, brain regions, and network labels for the corresponding components, uncorrected p-values (p_uncorr._), FDR-corrected p-values (p_FDR_), *T*-values and Cohen’s effect size (d). *EDSS: Expanded disability status scale; FDR: false discovery rate; BG: basal ganglia; ANG: angular gyrus; LOcc: Lateral occipital cortex, superior division; ANG: angular gyrus; CB: cerebellum; dATT: dorsal attention network; DMN: default mode network; SMN: somatomotor network.*

a Component region labels are bilateral unless otherwise noted.

**Table 6:** Dynamic functional connectivity results for comparison between healthy participants and patients with EDSS ≥ 2.

**State**	**Regions**^a^	**Networks**	**Components**	**p_uncorr._**	**p_FDR_**	**T**	**d**
1	Frontal pole – PCG	FPN – vATT	11 – 45	< 0.001	0.217	−3.729	−0.769
	STG – Precentral gyrus	DMN – SMN	30 – 37	< 0.001	0.451	−3.101	−0.656
2	Precentral gyrus – Precentral gyrus (right)	SMN – SMN	37 – 12	< 0.001	0.145	−3.387	−0.757
	ANG (right) – Precentral gyrus	DMN – SMN	53 – 37	< 0.001	0.115	−3.559	−0.761
	MFG (right) – Postcentral gyrus	FPN – SMN	58 – 89	< 0.001	0.145	−3.210	−0.576
	TOFC – Lingual gyrus	VIS – VIS	88 – 81	< 0.001	0.145	−3.631	−0.733
	LOcc – Lingual gyrus	VIS – VIS	96 – 81	< 0.001	0.145	−3.210	−0.886
	CB – Lingual gyrus	CB – VIS	27 – 81	< 0.001	0.115	−3.838	−0.901
3	MFG – SMG	dATT – dATT	91 – 49	< 0.001	0.102	3.525	0.788
	Cuneal cortex – MFG	VIS – dATT	42 – 91	< 0.001	0.102	3.400	0.771
	Precentral gyrus – Precentral gyrus (right)	SMN – SMN	37 – 12	< 0.001	0.121	3.319	0.872
	Precentral gyrus – Precentral gyrus (left)	SMN – SMN	37 – 14	< 0.001	0.102	3.598	0.865
	Cuneal cortex – Precentral gyrus	VIS – SMN	42 – 37	< 0.001	0.050*	4.269	0.986
	ANG (right) – LOcc	DMN – VIS	31 – 96	< 0.001	0.102	−3.557	−0.798
4	STG – Precentral gyrus	DMN – SMN	30 – 37	< 0.001	0.134	4.006	1.034
	Lingual gyrus – Postcentral gyrus	VIS – SMN	81 – 89	< 0.001	0.134	−3.937	−1.060
5	Frontal pole – Precentral gyrus	DMN – SMN	50 – 5	< 0.001	0.207	3.800	0.648
	Precentral gyrus – Precentral gyrus (right)	SMN – SMN	37 – 12	< 0.001	0.207	3.616	0.952
	Precuneus cortex – Cuneal cortex	DMN – VIS	40 – 42	< 0.001	<0.001*	4.780	0.974

Table includes state labels, brain regions, and network labels for corresponding components, uncorrected p-values (p_uncorr_), FDR-corrected p-values (p_FDR_), *T*-values and Cohen’s effect size (d). *EDSS: Expanded disability status scale; FDR: false discovery rate; PCG: paracingulate gyrus; STG: superior temporal gyrus, posterior division; ANG: angular gyrus; MFG: middle frontal gyrus; ACG: anterior cingulate gyrus; SMA: supplementary motor area; TOCF: temporal occipital fusiform cortex; LOcc: Lateral occpital cortex, superior division; CB: cerebellum; SMG: supramarginal gyrus; FPN: fronto-parietal control network; vATT: ventral attention network; DMN: default mode network; SMN: somatomotor network; VIS: visual network; dATT: dorsal attention network.*

a Component region labels are bilateral unless otherwise noted.

**Table 7:** Dynamic functional connectivity results for comparisons between patients with EDSS ≤ 1 and patients with EDSS ≥ 2.

**State**	**Regions**^a^	**Networks**	**Components**	**p_uncorr._**	**p_FDR_**	**T**	**d**
1	SMG – SMG	FPN – dATT	23 – 49	< 0.001	0.357	−3.332	−0.730
	Postcentral gyrus – Precentral gyrus	SMN – SMN	89 – 37	< 0.001	0.357	−3.481	−0.913
	LOcc – SMA	FPN – SMN	69 – 68	< 0.001	0.357	−3.229	−0.639
2	LOcc – TOFC	VIS – VIS	96 – 88	< 0.001	0.270	3.626	0.747
3	Lingual gyrus – Precentral gyrus	VIS – SMN	81 – 37	< 0.001	0.371	3.547	0.755
4	LOcc – TOFC	VIS – VIS	96 – 88	< 0.001	<0.001*	4.485	0.911
5	MFG (left) – Planum Temporale	FPN – SMN	52 – 15	< 0.001	0.368	−3.500	−0.787
	MFG (left) – Precentral gyrus	FPN – SMN	52 – 37	< 0.001	0.368	−3.353	−0.950

Table includes state labels, brain regions, and network labels for the corresponding components, uncorrected p-values (p_uncorr._), FDR-corrected p-values (p_FDR_), *T*-values and Cohen’s effect size (d). *EDSS: Expanded disability status scale; FDR: false discovery rate; SMG: supramarginal gyrus; LOcc: Lateral occipital cortex, superior division; SMA: supplementary motor area; TOCF: temporal occipital fusiform cortex; MFG: middle frontal gyrus; FPN: fronto-parietal control network; dATT: dorsal attention network; SMN: somatomotor network; VIS: visual network.*

a Component region labels are bilateral unless otherwise noted.

#### 3.2.3 Dynamic metric group differences

**Fig. 4:**
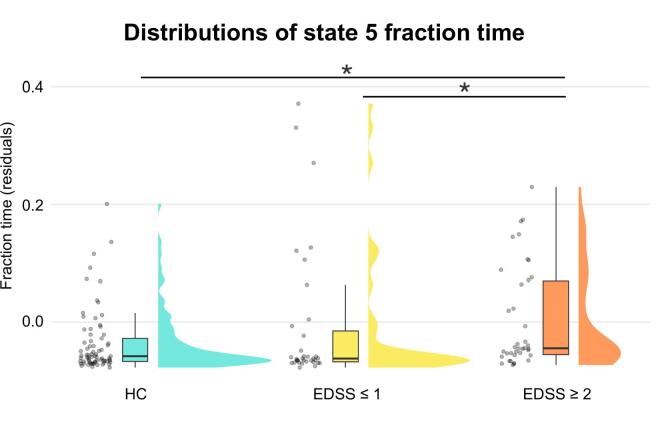
Group differences in state five fraction time. Raincloud plots show group-wise distributions of state five fraction time residuals after age-related variance was regressed out. Patients with mild to moderate levels of disability (EDSS ≥ 2) spent significantly more time in state 5 compared to patients without disability (EDSS ≤ 1) and HCs. Outliers have been removed for box-plot visualization purposes. Asterisks indicate significance level: * = p_FDR_ < 0.05.

No group effects were observed for average connectivity, modularity, or dwell time. In contrast, a significant group effect was present for state 5 stickiness (i.e., the number of times a participant remained in state 5 over two consecutive windows) as well as for transitions between states 3–5 and 4–5. Further analyses showed that this effect was due to differences in how often this state was visited. Accordingly, we observed a significant group effect on state 5 fraction time (i.e., the proportion of total scan duration a participant spent in state 5), such that patients with mild to moderate disability spent significantly more time in state 5 than both patients without disability and HCs (Table 8; Fig. 4).

**Table 8:** Group differences in state five fraction time after age regression.

Kruskal-Wallis test			Dunn’s test			
χ^2^	df	p	Comparisons	Z	p_uncorr._	p_FDR_
7.87	2	0.019*	HC vs EDSS ≤ 1	0.097	0.923	0.923
			HC vs EDSS ≥ 2	−2.612	0.009	0.027*
			EDSS ≤ 1 vs EDSS ≥ 2	−2.316	0.021	0.031*

Table includes test statistics and p-values from the omnibus Kruskal-Wallis test and the Dunn’s test for pairwise comparisons. Significant results are marked with an asterisk. *Df: degrees of freedom; FDR: false discovery rate; HC: healthy control participants; MS EDSS ≤ 1: MS patients with expanded disability status scale score less than or equal to 1; MS EDSS ≥ 2: MS patients with expanded disability status scale score greater than or equal to 2.*

#### 3.2.4 Across-state overall connectivity

**Fig. 5:**
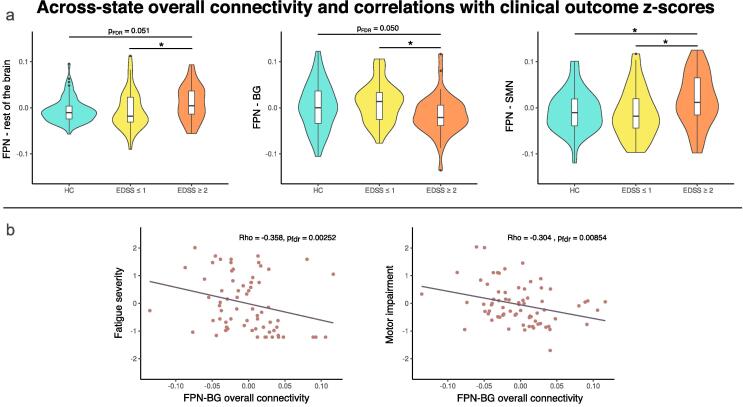
Group differences and correlations with frontoparietal across-state overall connectivity. Panel a: (Left) Patients with mild to moderate disability have higher across-state overall connectivity (ASOC) between the frontoparietal network (FPN) and the rest of the brain compared to patients with no disability; (Middle) Patients with mild to moderate disability have lower ASOC between the FPN and basal ganglia (BG) compared to patients with no disability, with a similar trend compared to HCs; (Right) Patients with mild to moderate disability have higher ASOC between the SMN) compared to both patients with no demonstrated disability and HC. Panel b: FPN overall connectivity with the BG is negatively correlated with fatigue (left) and motor impairment (right); Asterisks denote significance level: * = p_FDR_ < 0.05.

Among all participants, state 5 had the highest average connectivity. We also observed that patients with mild to moderate disability spent more time in this high-connectivity state compared to both patients without disability and HCs. Thus, we hypothesized that patients in the mild to moderate disability group would also display higher global average connectivity across all states. In other words, spending more time in the highest connectivity state would drive a parallel increase in connectivity when considering all states simultaneously. On a global scale, we observed a gradual increase in ASOC from HC to patients with EDSS ≤ 1 to patients with EDSS ≥ 2. However, this was limited to a statistical trend. On the network scale, a significant group effect was found on inter-network connectivity between the FPN and the rest of the brain, such that patients with EDSS ≥ 2 had significantly higher FPN inter-network ASOC than patients with EDSS ≤ 1 and a trend in the same direction compared to HC. We then further investigated which inter-network connections may drive this result. Fig. 5 shows that patients with mild to moderate disability had significantly higher FPN-SMN ASOC than patients with no disability and HCs, as well as significantly lower FPN-BG ASOC compared to patients with no disability and a similar trend compared to HCs (Table 9). Correlation analyses between EDSS scores and FPN-BG overall connectivity strength revealed a significant negative association (rho = -0.3844, p = < 0.001; Supplementary Fig. 5). Thus, an inverse relationship between disability and FPN-BG ASOC was observed on both the group-level and when considering the distribution of EDSS scores across the patient sample. Critically, FPN-BG connectivity was also negatively correlated with fatigue and motor impairment (Fig. 5b). Control analyses using static FC data to estimate overall connectivity corroborated these findings. However, in these static analyses, group effects on overall connectivity between the FPN and the rest of the brain were not significant. Additional details are provided in SM section 2.2.

**Table 9:** Between-group differences in frontoparietal network across-state overall connectivity.

Networks	Kruskal-Wallis test			Dunn’s test			
	χ^2^	df	p	Group comparisons	Z	p_uncorr._	p_FDR_
FPN - rest of the brain	6.64	2	0.036*	HC vs EDSS ≤ 1	0.675	0.499	0.499
				HC vs EDSS ≥ 2	−2.120	0.034	0.051
				EDSS ≤ 1 vs EDSS ≥ 2	−2.404	0.016	0.049*
FPN - SMN	7.25	22	0.027*	HC vs EDSS ≤ 1	0.588	0.556	0.556
				HC vs EDSS ≥ 2	−2.283	0.022	0.034*
				EDSS ≤ 1 vs EDSS ≥ 2	−2.466	0.014	0.041*
FPN - BG	7.52	22	0.023*	HC vs EDSS ≤ 1	−0.927	0.354	0.354
				HC vs EDSS ≥ 2	2.125	0.034	0.050*
				EDSS ≤ 1 vs EDSS ≥ 2	2.628	0.008	0.026*

Table shows the networks used for each test (Networks), test statistics and p-value for the Kruskal-Wallis omnibus tests, and test statistics and p-values for the Dunn’s tests for pairwise comparisons*; FPN: fronto-parietal control network; SMN: somatomotor network; BG: basal ganglia; Df: degrees of freedom; FDR: false discovery rate; HC: healthy control participants; MS EDSS ≤ 1: MS patients with expanded disability status scale score less than or equal to 1; MS EDSS ≥ 2: MS patients with expanded disability status scale score greater than or equal to 2.*

### 3.3 Associations with clinical impairment

**Fig. 6:**
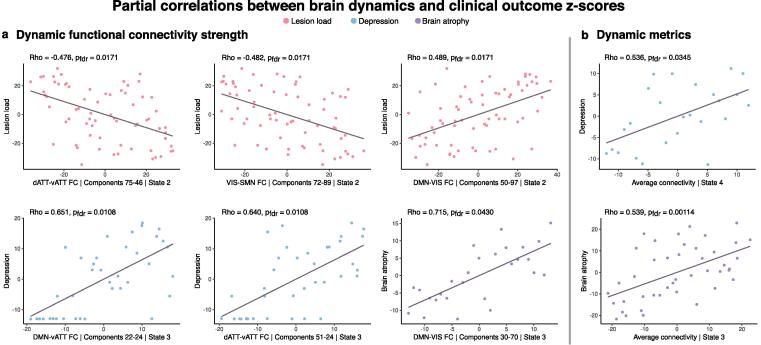
Partial correlations between dynamic functional connectivity, dynamic metrics, and clinical outcome z-scores. Panel a: Whole-brain dynamic functional connectivity values across patients were correlated with domain impairment scores. X-axes of subplots denote the resting-state network assignment, component numbers, and state in which the corresponding functional connection was significantly related to patient impairment indicated by the y-axis labels. Panel b: Dynamic metrics (x-axes) were correlated with domain impairment scores (y-axes). All correlations were performed using Spearman’s partial correlations controlling for age, and false discovery rate (FDR) correction was applied within state and domain. Values plotted correspond to variable residuals after rank-based age regression.

Fig. 6 shows the results of correlation analyses between dFC, dynamic metrics, and clinical outcome scores that persisted when controlling for age. Lesion load was negatively correlated with both dATT-vATT and VIS-SMN connectivity in state 2. In contrast, lesion load was positively correlated with DMN-VIS connectivity in state 2. Depression was positively correlated with both DMN-vATT and dATT-vATT connectivity in state 3. Finally, total brain atrophy was positively correlated with DMN-VIS connectivity in state 3. Correlation analyses between dynamic metrics and clinical scores revealed that depression severity was positively correlated with state 4 average connectivity, and brain atrophy was positively correlated with average connectivity of state 3.

Importantly, correlations between static FC strength and clinical scores were not significant. Similarly, no relationship was found between static global average connectivity and any domain clinical score. However, static modularity was positively correlated with brain atrophy (rho = 0.293, p_FDR_ = 0.037).

## 4 Discussion

In the present study, we show that functional brain dynamics in RRMS exhibit widespread alterations that vary with disability status and reflect clinical outcomes across multiple symptom domains – even in a sample of patients with early-stage MS who show low levels of disability overall. Categorizing patients based on EDSS ratings revealed consistently higher impairment across all clinical domains in patients with mild to moderate disability compared to those without disability, supporting the validity of our approach with respect to clinical outcomes. Despite the overall low disease severity and the early disease stage, we reveal distinct patterns of altered connectivity between patients with without clinical disability and those with mild to moderate disability. With our whole-brain dynamic FC approach, we observed widespread bidirectional FC alterations across cortical and subcortical components in MS patients. While dynamic analyses revealed an intricate pattern of inter- and intra-network alterations, static analyses only detected inter-network changes. These observations suggest that sFC approaches may obscure transient intra-network connectivity changes that are observed only on the finer temporal scales of time-resolved analyses (i.e., seconds versus minutes). Furthermore, we observed significant relationships between functional connectivity, depressive symptoms, and structural brain measures in patients that remained undetected with a static approach. These findings highlight the advantages of time-resolved methods in studying functional network interactions and emphasize their role in understanding the complex relationships between functional brain dynamics, disability status, and clinical outcomes in MS.

Regarding the implicated brain systems, we found consistent implication of the frontoparietal and default mode networks in component-specific FC differences between patients with mild to moderate disability and patients without disability in both static and dynamic analyses. In the case of static FC, patients with mild to moderate disability showed predominantly increased FC between FPN, DMN and several other networks compared to patients without disability. On the other hand, dynamic analyses revealed a widespread pattern of connectivity alterations between patient groups, such that FPN connections to other RSNs were predominantly increased and DMN inter-network connectivity strength decreased in patients with mild to moderate disability compared to those without clinical disability. Altered FC in the FPN and DMN have been reported in previous studies of patients with MS. For example, increased static FC of core FPN and DMN regions was associated with loss of cognitive efficiency (Hawellek et al., 2011). Relatedly, another recent study reported increased connectivity between DMN and FPN to the rest of the brain in cognitively impaired patients with MS (Meijer et al., 2017). Static FC results of the present study support these previous findings. However, age-controlled correlations between clinical scores and static FC did not yield any significant associations in this study. In the case of dynamic FC, two previous studies have applied a similar methodological approach to studying whole-brain connectivity states in MS as used here. Cognitively impaired patients with relapsing-remitting MS showed reduced dynamism of resting-state FC compared to cognitively preserved patients, specifically between subcortical and default mode networks (d’Ambrosio et al., 2020). Reduced dFC in sensorimotor, cerebellar and cognitive networks was also identified across the main subtypes of MS, with worsening FC abnormalities correlated to motor and cognitive impairment only apparent in progressive MS (Hidalgo de la Cruz et al., 2021). While these studies also implicated subcortical, default mode, and attention networks, they focused on cognitive impairment in RRMS and relationships between disability and symptom severity across a sample of patients with high clinical variability, respectively. Against this background, a major contribution of the present work is the identification of distinct alterations in FC dynamics between two groups of RRMS patients who are both in early stages of the disease and have overall low levels of disability. Despite a relatively narrow range of EDSS scores in the present population, we show that impairment in cognitive, behavioral, and neuropsychological domains as well as structural brain atrophy is associated with higher disability ratings and relates to several measures of functional brain dynamics, even early on in the disease.

We observed that increased depression symptoms were positively correlated with FC between the vATT-DMN and vATT-dATT in patients with MS. Importantly, the relevant DMN component corresponded to the bilateral hippocampus, which has been repeatedly implicated in depressive symptoms (Sheline et al., 2002) and shown to be affected in MS (Heine et al., 2020, Rocca et al., 2018). In a study of hippocampal neuroinflammation, connectivity, and depression symptoms in MS patients, both positive and negative relationships between hippocampal resting-state connectivity and depression scores were reported (Colasanti et al., 2016). While our results relating to hippocampal connectivity complement these findings, we furthermore identify a potential role of ventral and dorsal attention networks in MS-related depressive symptoms. This relationship between depression and FC increase is further supported by the positive association between depressive symptoms and global average connectivity in state 4. Taken together, these findings show that transient increases in FC, both globally and network-specific, are related to depressive symptom severity in patients with MS. The lack of this relationship in static connectivity analyses suggests that it may be specifically altered temporal brain dynamics that play a role in depressive symptoms in MS. Looking to evidence from studies of patients with major depression, alterations in functional dynamics across several RSNs, namely the default mode, frontoparietal, limbic, and salience networks have been consistently related to symptom severity in multiple domains of depression, including negative thinking and rumination, as well as overall disease severity (Kaiser et al., 2016, Marchitelli et al., 2022). Thus, these findings further point to an essential role of temporal dynamics in understanding the link between brain network behavior and clinical presentation.

Fatigue and motor impairment are common features of MS. Several studies have reported inverse relationships between DMN-BG FC alterations and fatigue in MS. With a seed-based sFC approach, a negative relationship between fatigue severity and FC of BG with typical DMN structures has been observed (Finke et al., 2015, Jaeger et al., 2019). More recently, a dynamic approach has similarly reported that lower FC between the BG and DMN was associated with greater fatigue in patients with MS (Tijhuis et al., 2021). Here, we likewise observe the involvement of the BG, but instead implicate its connections to the FPN.

We found that decreased overall connectivity between the FPN and BG was systematically associated with higher motor performance impairment, higher fatigue severity scores, and higher EDSS ratings in our patient cohort. While the implication of BG dysfunction and atrophy is well-documented in MS, specific FPN-BG interactions in this population are less understood. In healthy participants, the role of BG in motor control is well-established (Brooks, 1995). Additionally, the FPN is typically regarded as a task-positive network involved in cognitive control and facilitating coordination between other brain networks (Marek and Dosenbach, 2018). With this context, the observed consistent relationship between FPN-BG connectivity and clinical impairment across multiple domains suggests a clinically relevant functional decoupling between the FPN and BG in MS, pointing to an exciting new brain target linking aberrant FC to clinical outcomes.

### 4.1 Limitations

Some limitations of the present work warrant mentioning. We analyzed a cross-sectional sample of data, which places an inherent limitation on investigating causal mechanisms of observed links between FC, disability, and clinical impairment. Additionally, neuropsychological and behavioral data were not sufficiently available for HCs and were thus not included in between-group analyses. This prevented us from making direct comparisons between patient subgroups and HCs when analyzing clinical outcome scores and limited the interpretation of impairment severity against normative values. Additionally, variance in current medication across the patient sample was not considered in statistical analyses. Thus, it is not possible to exclude a potential influence of medication on the observed differences in functional brain dynamics or clinical outcome scores. Finally, it is important to note that while clustering-based analysis of brain states represents one of the most well-studied approaches to describe functional brain dynamics (Allen et al., 20142014, Calhoun et al., 2001, Calhoun et al., 2014), other approaches such as eigenvector centrality or whole-brain modelling are increasingly explored and might yield additional information in describing functional alterations in early MS. Finally, here we discretized the distribution of EDSS scores across the sample to obtain one group of patients with mild to moderate disability and one group of patients without disability. While further work is necessary to obtain more continuous accounts of the link between FC aberrations and clinical impairment, we also show in rank-based correlation analyses that the association between multi-domain clinical outcomes and EDSS scores holds on an ordinal scale.

## 5 Conclusions

These results show that connectivity alterations in early MS are not homogeneous but depend on temporal network dynamics and level of disability, even in a sample of patients with comparably low overall disability status. With multi-domain clinical outcome scores, we show that – even in early stages of the disease – it is possible to identify significant differences in depressive and fatigue symptom severity, cognitive and motor performance, visual acuity, lesion load, and total brain atrophy between patients with no clinical disability and those with mild to moderate levels of disability according to EDSS. Despite the overall low disability in this population, we reveal distinct connectivity alterations between patient groups that are meaningful for clinical outcomes. Notably, the FPN was consistently implicated in both FC group differences and clinical associations to fatigue and motor performance impairment. Finally, dynamic analyses revealed both FC alterations and behavioral correlations that remained undetected in static analyses, highlighting that temporal brain dynamics are important to help disentangle the relationship between functional alterations, disability, and clinical outcomes in early MS.

## Funding

AR is a doctoral candidate at the Berlin School of Mind and Brain, funded by the Humboldt-Universität zu Berlin. SK was funded by the Deutsche Forschungsgemeinschaft (DFG, German Research Foundation), grant number FI 2309/2–1. NS is a doctoral scholar at Cusanuswerk – Bischöfliche Studienförderung. CC receives funding through Novartis and the Bundesministerium für Bildung und Forschung. KR is a participant in the BIH Clinical Fellow Program funded by Stiftung Charité. FP receives research support from Bayer, Novartis, Biogen Idec, Teva, Sanofi-Aventis / Genzyme, Alexion and Merck Serono, German Research Council (DFG Exc 257), Werth Stiftung of the City of Cologne, German Ministry of Education and Research (BMBF Competence Network Multiple Sclerosis), Arthur Arnstein Stiftung Berlin, EU FP7 Framework Program (combims.eu), Guthy Jackson Charitable Foundation, and National Multiple Sclerosis Society of the USA. CF was funded by the Deutsche Forschungsgemeinschaft (DFG, German Research Foundation) grant numbers FI 2309/1–1 (Heisenberg Program), FI 2309/2–1 and 327654276 (SFB 1315); and the German Ministry of Education and Research (BMBF) grant number 01GM1908D (CONNECT-GENERATE).

## CRediT authorship contribution statement

**Amy Romanello:** Conceptualization, Methodology, Validation, Formal analysis, Visualization, Writing – original draft, Writing – review & editing. **Stephan Krohn:** Conceptualization, Methodology, Formal analysis, Supervision, Writing – original draft, Writing – review & editing. **Nina von Schwanenflug:** Conceptualization, Methodology, Validation, Formal analysis, Writing – original draft, Writing – review & editing. **Claudia Chien:** Formal analysis, Investigation, Data curation, Writing – review & editing. **Judith Bellmann-Strobl:** Investigation, Writing – review & editing. **Klemens Ruprecht:** Investigation, Writing – review & editing. **Friedemann Paul:** Resources, Supervision, Funding acquisition, Writing – review & editing. **Carsten Finke:** Conceptualization, Resources, Supervision, Funding acquisition, Writing – original draft, Writing – review & editing.

## Declaration of Competing Interest

The authors declare the following financial interests/personal relationships which may be considered as potential competing interests: AR, SK, NS, CF have nothing to disclose. CC has received speaking honoraria from Bayer and research funding from Novartis, unrelated to this study. JBS has received speaking honoraria and travel grants from Bayer Healthcare and sanofi-aventis/Genzyme, as well as compensation for serving on a scientific advisory board of Roche, unrelated to this study. KR received research support from Novartis Pharma, Merck Serono, German Ministry of Education and Research, European Union (821283–2), Stiftung Charité (BIH Clinical Fellow Program) and Arthur Arnstein Foundation; received travel grants from Guthy Jackson Charitable Foundation. FP serves on the Novartis OCTIMS study steering committee and the MedImmune / Viela Bio steering committee; received speaker honoraria and travel grants from Bayer, Novartis, Biogen Idec, Teva, Sanofi-Aventis / Genzyme, and Merck Serono, Alexion, Chugai, MedImmune, Shire, Roche, Actelion, Celgene; consultancies for SanofiGenzyme, BiogenIdec, MedImmune, Shire, Alexion.

## Data Availability

The data that has been used is available upon reasonable request to the corresponding author.
